# Association Between Obesity and Poor Prognosis in Patients Receiving Anlotinib for Advanced Non-Small Cell Lung Cancer

**DOI:** 10.3389/fphar.2022.812555

**Published:** 2022-03-30

**Authors:** Anning Xiong, Wei Nie, Lei Cheng, Hua Zhong, Tianqing Chu, Runbo Zhong, Jun Lu, Shuyuan Wang, Jianlin Xu, Yinchen Shen, Feng Pan, Baohui Han, Xueyan Zhang

**Affiliations:** Department of Pulmonary, Shanghai Chest Hospital, Shanghai Jiao Tong University, Shanghai, China

**Keywords:** VEGFR-TKIs, body mass index, anti-angiogenesis, predictive biomarker, multi-targer drug

## Abstract

**Background:** Anlotinib is a novel anti-angiogenesis drug. In non-small cell lung cancer (NSCLC), high body mass index (BMI) was not associated with worse survival in patients treated with bevacizumab compared with those with normal or low BMI. However, it remains unknown whether such an association still exists in NSCLC patients receiving anlotinib therapy. Hence, we conducted this study to investigate whether BMI is associated with clinical outcomes in patients treated with anlotinib for advanced NSCLC.

**Methods:** Data of 554 patients from the ALTER-0302 and the ALTER-0303 trials were analyzed in this study. The patients were classified into non-obesity (BMI <28 kg/m^2^) and obesity (BMI ≥28 kg/m^2^) subgroups. The primary endpoint was overall survival (OS). The secondary endpoints included progression-free survival (PFS), objective response rate (ORR), and disease control rate (DCR). OS was defined as the interval between the first drug administration and death. PFS was defined as the time span from the date of initiating the treatment to the first documented progression or death from any cause, whichever occurred first. ORR included complete response (CR) and partial response (PR).

**Results:** There were 354 patients (63.9%) who received anlotinib in this study. Restricted cubic spline model showed a U-shaped relation between BMI and the risk of death in the anlotinib group. In a multivariable Cox regression model, a trend of worse overall survival was observed in obese patients who received anlotinib compared with placebo (HR, 2.33; 95% CI, 0.77–7.06; *p* = 0.136). The interaction between BMI stratification and treatment was significant for OS (P for interaction = 0.038).

**Conclusion:** Our results revealed a U-shaped relationship between BMI and risk of death in patients receiving anlotinib for advanced NSCLC. More importantly, obesity (BMI ≥28 kg/m^2^) might be a potential predictor of use of anlotinib in advanced NSCLC.

## Background

Obesity is widely considered as a poor prognostic factor in cancer (Lennon et al., 2016). However, the relationship between obesity and favorable prognosis has been reported in patients with early or advanced lung cancer who received different treatment including surgery, chemotherapy, and radiotherapy (Dahlberg et al., 2013; Yap et al., 2018; Icard et al., 2020), which is called the “obesity paradox” (Zhang et al., 2017). This phenomenon was seen in lung cancer patients treated with tyrosine kinase inhibitor (TKI) before. A retrospective study has demonstrated that patients with a body mass index (BMI) ≥ 25 kg/m^2^ had longer progression-free survival (PFS) and overall survival (OS) compared with those with BMI <18.5 kg/m^2^ when they received epidermal growth factor receptor (EGFR-TKI) for non-small cell lung cancer (NSCLC). Moreover, BMI was an independent predictor of outcomes in this study (Park et al., 2016). In addition, a trend to prolonged PFS and OS was observed in advanced NSCLC patients with EGFR-TKI therapy and with higher body weight, though it was not significant (Imai et al., 2017).

As a novel TKI, anlotinib suppresses tumor proliferation by inhibiting tumor angiogenesis (Sun et al., 2016). Due to the results of the ALTER-0303 trial, anlotinib has been approved as a third-line or further therapy for advanced NSCLC in China (Zhou et al., 2019). Nevertheless, the objective response rate (ORR) of anlotinib was about 10% and adverse events occurred more frequently in patients treated with anlotinib than placebo (Han et al., 2018a). Therefore, great efforts have been made to find potential predictive biomarkers so that patients who are more likely to response to anlotinib could be identified earlier (Lu et al., 2019a; Lu et al., 2019b; Lu et al., 2019c). However, these predictors are relatively complex and expensive to use in clinical practice. Thus, researchers are trying to find simple and convenient predictive factors of anlotinib use.

BMI is a readily available index to measure weight. A previous meta-analysis has reported that no association is observed between obesity and worse clinical outcomes compared with normal or low BMI in patients treated with bevacizumab for advanced NSCLC (Shukla et al., 2021). However, it remains unknown whether there is a relationship between obesity and poor clinical outcomes in patients receiving anlotinib for NSCLC.

Therefore, we conducted this study to investigate whether BMI is associated with clinical outcomes in patients treated with anlotinib for advanced NSCLC.

## Methods

### Patients

**FIGURE 1:**
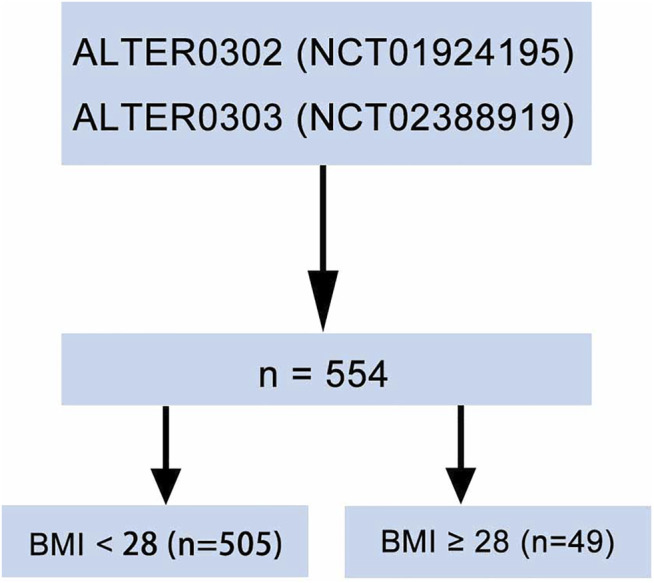
Diagram of patient inclusion. Body mass index (BMI).

A total of 554 patients with evaluable data were obtained from the ALTER-0302 (Han et al., 2018a) and the ALTER-0303 (Han et al., 2018b) trials (Figure 1). The number of the ethical approval was LS1504. All the researchers have agreed on this study. These were multicenter, double-blind, randomized clinical trials that investigated the efficacy of anlotinib as a third-line or further treatment in patients with advanced NSCLC. Patients were randomly assigned to receive either anlotinib or placebo in a 1:1 ratio. Oral anlotinib was administrated with a dosage of 12 mg per day on Days 1–14 of a 21-d cycle. Informed consent was obtained from all patients in the two studies.

The trials were conducted according to the principles of the International Conference of Harmonisation Good Clinical Practice, the Declaration of Helsinki, and local institutional review board requirements.

### Endpoints

The primary endpoint was OS. The secondary endpoints included PFS, ORR, and disease control rate (DCR). OS was measured from the date of randomization to the date of death from any cause or the last follow-up. PFS was defined as the time span between randomization to disease progression or mortality due to any cause, which occurred first. ORR was measured as the proportion of patients experiencing a complete response (CR) or a partial response (PR) as the best response to therapy. DCR consisted of CR, PR, and stable disease (SD). Tumor response was assessed according to the Response Evaluation Criteria in Solid Tumors, version 1.1.

### Statistical Analysis

Associations between BMI and OS were flexibly modeled using restricted cubic spline (RCS) curves based on Cox proportional hazards models in anlotinib and placebo treatment groups. These analyses helped to define a reference BMI value. A reference value of 28 was chosen due to the BMI standard in China (China, 2004) and patients were subsequently stratified into non-obesity group (BMI <28 kg/m^2^) and obesity group (BMI ≥28 kg/m^2^). The differences of baseline characteristics between the two groups were examined using Pearson’s χ^2^ test. χ^2^ test was also used to compare ORR and DCR between non-obesity and obesity groups. The Kaplan-Meier method and log-rank test were used to analyze OS and PFS. The hazard ratios (HRs) of OS and PFS were estimated by utilizing the Cox proportional hazards model. Stratified Cox model containing variables of therapy, BMI, and treatment by BMI interaction were used to evaluate the *p* value for interaction. All *p* values were two sided and a *p* value < 0.05 was considered statistically significant. Data analysis was carried out using R, version 3.6.1 (R Project for Statistical Computing) and SPSS version 25.0 (IBM, Armonk, NY).

## Results

### Baseline Characteristics

**FIGURE 2:**
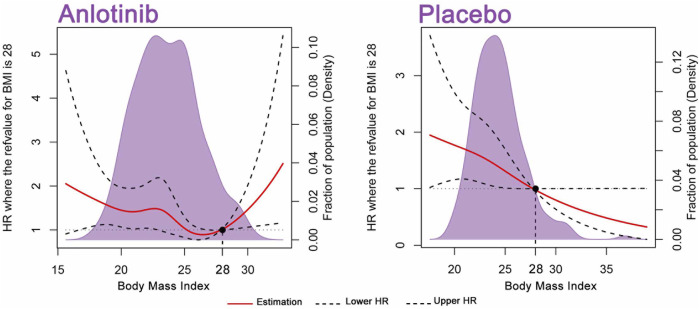
BMI on a continuous scale and the risk of mortality in 554 patients. Analyses were conducted using restricted cubic splines, with hazard ratios and 95% confidence intervals from multiple-event Cox proportional hazards regression. The BMI value of 28 was chosen as a reference. The purple areas indicate the distribution of concentrations of BMI. Body mass index (BMI).

A total of 554 patients were included in this study. In Figure 2, RCS was developed to smoothly model and display the relation between BMI and HR for OS. According to the BMI standard in China, 28 was chosen as a reference value. In patients receiving placebo, the RCS model showed a negative relation between BMI and the risk of mortality. High BMI was associated with low risk of death, which is in accordance with the obesity paradox. Regarding the relation between BMI and the risk of mortality in the anlotinib treatment group, a U-shaped association was seen. The risk of mortality decreased until around the BMI value of 28 and then turned to increase afterward. Above 28, the HRs of mortality related to BMI values elevated sharply.

Due to the relationship between BMI and the risk of mortality depicted above, the 554 patients were classified into non-obesity (BMI <28 kg/m^2^) and obesity (BMI ≥28 kg/m^2^) subgroups. The mean values of BMI were 22.8 kg/m^2^ (15.6–27.9) and 30.0 kg/m^2^ (28.1–39.0) in the non-obese and obese groups, respectively. There were 49 patients (8.8%) in the obesity group. Baseline demographic and clinical characteristics were balanced between non-obesity and obesity subgroups except for Eastern Cooperative Oncology Group (ECOG) Performance Status (PS) score and lines of previous chemotherapy. Obese patients had lower PS score (*p* = 0.005) and received more lines of previous chemotherapy (*p* = 0.006) (Table 1).

**TABLE 1:** Baseline characteristics of the patients.

Characteristic	Non-obesity (*n* = 505)	Obesity (*n* = 49)	*P*
Age (years) (%)			
≤60	302 (59.8)	29 (59.2)	0.933
>60	203 (40.2)	20 (40.8)	
Gender (%)			
Male	317 (62.8)	27 (55.1)	0.291
Female	188 (37.2)	22 (44.9)	
Smoking history (%)			
Never	257 (50.9)	31 (63.3)	0.098
Current/former	248 (49.1)	18 (36.7)	
ECOG (%)			
0	76 (15.0)	15 (30.6)	**0.005**
1–2	429 (85.0)	34 (69.4)	
Histology (%)			
Adenocarcinoma	415 (82.2)	42 (85.7)	0.534
Squamous	90 (17.8)	7 (14.3)	
Stage (%)			
Ⅲ	29 (5.8)	1 (2.0)	0.443
Ⅳ	474 (94.2)	48 (98.0)	
EGFR mutation (%)			
Positive	147 (29.1)	12 (24.5)	0.776
Negative	295 (58.4)	30 (61.2)	
Unknown	63 (12.5)	7 (14.3)	
Number of metastases (%)			
≤3	259 (51.3)	30 (61.2)	0.184
>3	246 (48.7)	19 (38.8)	
Treatment (%)			
Anlotinib	318 (63.0)	36 (73.5)	0.144
Placebo	187 (37.0)	13 (26.5)	
Lines of previous chemotherapy (%)			
1–2	281 (55.6)	19 (38.8)	**0.006**
≥3	224 (44.4)	29 (59.2)	
Unknown	0 (0)	1 (2.0)	
Previous			
Targeted treatment (%)			
Yes	217 (43.0)	29 (59.2)	0.771
No	288 (57.0)	20 (40.8)	
Radiotherapy history (%)			
Yes	204 (40.4)	18 (36.7)	0.618
No	301 (59.6)	31 (63.3)	

The bold values mean that the p value was considered statistically significant. Abbreviations: Eastern Cooperative Oncology Group (ECOG); epidermal growth factor receptor (EGFR).

### Primary and Secondary Outcomes

In the non-obesity group, multivariate analysis demonstrated that anlotinib therapy (*p* = 0.002), PS score of 0 (*p* = 0.005), EGFR mutation (*p* = 0.022), number of metastases ≤3 (*p* < 0.001), and less lines of previous chemotherapy (*p* = 0.013) were independent predictors of improved OS (Table 2).

**TABLE 2:** Univariate and multivariable analyses for variables associated with OS in non-obesity (*n* = 505).

Characteristics	Category	Univariate analysis HR (95%CI)	*p*	Multivariate analysis HR (95%CI)	*p*
Treatment	Anlotinib vs. placebo	0.704 (0.577–0.858)	**0.001**	0.706 (0.568–0.877)	**0.002**
Age	>60 vs. ≤60	1.103 (0.907–1.341)	0.326	1.132 (0.911–1.406)	0.264
Gender	Male vs. female	1.461 (1.194–1.789)	**<0.001**	1.213 (0.906–1.624)	0.195
Smoking history	Current/former vs. never	1.420 (1.169–1.724)	**<0.001**	1.208 (0.920–1.586)	0.174
Stage	Ⅳ vs. Ⅲ	1.286 (0.836–1.977)	0.252	1.125 (0.683–1.852)	0.644
ECOG	1–2 vs. 0	1.509 (1.138–2.002)	**0.004**	1.542 (1.143–2.081)	**0.005**
Histology	Squamous vs. adenocarcinoma	1.106 (0.862–1.418)	0.429	0.947 (0.714–1.256)	0.704
EGFR mutation	Positive vs. negative	0.717 (0.574–0.896)	**0.003**	0.717 (0.540–0.952)	**0.022**
Number of metastases	>3 vs. ≤3	1.573 (1.297–1.908)	**<0.001**	1.596 (1.291–1.974)	**<0.001**
Lines of previous chemotherapy	≥3 vs. 1–2	0.810 (0.667–0.985)	**0.034**	0.760 (0.612–0.944)	**0.013**
Previous Targeted treatment	Yes vs. no	0.828 (0.682–1.005)	0.056	1.019 (0.787–1.319)	0.886
Radiotherapy history	Yes vs. no	1.153 (0.949–1.401)	0.153	1.151 (0.929–1.427)	0.199

The bold values mean that the p value was considered statistically significant. Abbreviations: Eastern Cooperative Oncology Group (ECOG); epidermal growth factor receptor (EGFR).

In the obesity group, only the number of metastases >3 (*p* = 0.033) was an independent factor of poor OS (Table 3).

**TABLE 3:** Univariate and multivariable analyses for variables associated with OS in the obesity group (*n* = 49).

Characteristics	Category	Univariate analysis HR (95%CI)	*p*	Multivariate analysis HR (95%CI)	*p*
Treatment	Anlotinib vs. placebo	1.792 (0.758–4.233)	0.184	2.326 (0.766–7.057)	0.136
Age	>60 vs. ≤60	0.634 (0.317–1.268)	0.198	0.463 (0.181–1.184)	0.108
Gender	Male vs. female	0.878 (0.460–1.676)	0.694	0.462 (0.130–1.647)	0.234
Smoking history	Current/former vs. never	1.149 (0.586–2.254)	0.686	2.843 (0.827–9.771)	0.097
ECOG	1–2 vs. 0	0.975 (0.492–1.930)	0.942	1.057 (0.462–2.419)	0.896
Histology	Squamous vs. adenocarcinoma	2.227 (0.911–5.449)	0.079	2.580 (0.688–9.677)	0.160
EGFR mutation	Positive vs. negative	1.095 (0.501–2.397)	0.820	1.551 (0.555–4.336)	0.402
Number of metastases	>3 vs. ≤3	1.575 (0.823–3.013)	0.170	2.542 (1.079–5.987)	**0.033**
Lines of previous chemotherapy	≥3 vs. 1–2	0.857 (0.441–1.665)	0.649	0.879 (0.374–2.064)	0.767
Previous Targeted treatment	Yes vs. no	1.069 (0.556–2.057)	0.841	0.536 (0.179–1.601)	0.264
Radiotherapy history	Yes vs. no	1.652 (0.863–3.163)	0.130	2.046 (0.821–5.096)	0.124

The bold values mean that the p value was considered statistically significant. Abbreviations: Overall survival (OS); hazard ratio (HR); Eastern Cooperative Oncology Group (ECOG); epidermal growth factor receptor (EGFR).

PFS was significantly longer in patients treated with anlotinib compared with placebo in both non-obesity (*p* < 0.001) and obesity (*p* = 0.025) groups. Anlotinib was also significantly associated with significantly higher DCR in the non-obesity (*p* < 0.001) and obesity (*p* = 0.046) groups. Moreover, anlotinib treatment resulted in significantly higher ORR in the non-obesity group (*p* < 0.001) (Supplementary Tables S1 and S2).

### Obesity (BMI ≥28 kg/m^2^) Predicted Short OS in Patients Receiving Anlotinib

**FIGURE 3:**
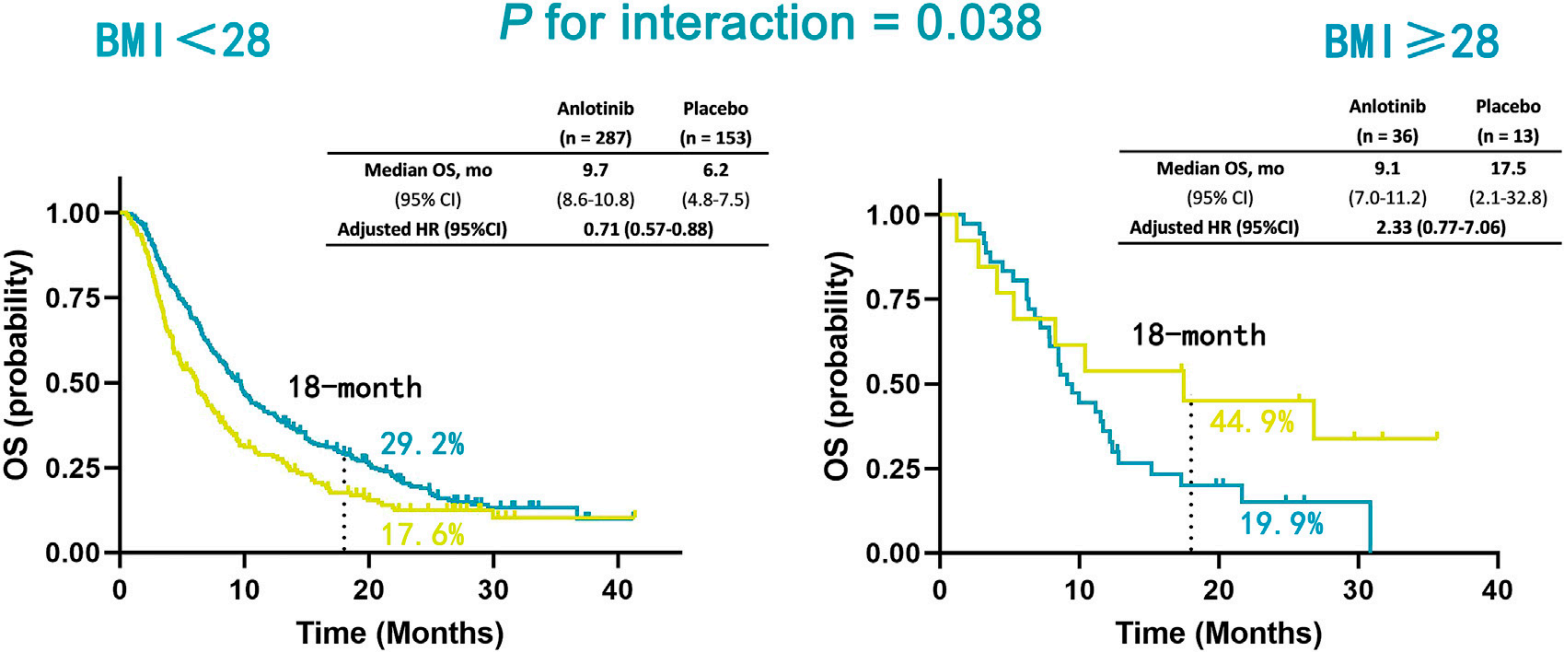
Kaplan-Meier estimates of OS in non-obesity (BMI <28 kg/m^2^) and obesity (BMI ≥28 kg/m^2^) patients in anlotinib and placebo treatment groups. Overall survival (OS); body mass index (BMI).

In the non-obesity group (BMI <28 kg/m^2^), patients treated with anlotinib had a significantly improved OS compared with placebo (adjusted hazard ratio [HR], 0.71; 95% CI, 0.57–0.88; *p* = 0.002), while a trend of poorer OS was observed in patients receiving anlotinib compared with placebo (HR, 2.33; 95% CI, 0.77–7.06; *p* = 0.136). The interaction between BMI classification and treatment was significant for OS (P for interaction = 0.038; Figure 3).

## Discussion

In this study, a U-shaped association between BMI and risk of death was observed in patients receiving anlotinib for advanced NSCLC. An increased risk was observed in all BMI values equal to or above 28 kg/m^2^ for death. After classifying patients into non-obesity (BMI <28 kg/m^2^) and obesity (BMI ≥28 kg/m^2^) groups, we found that obesity was a predictor of worse efficacy of anlotinib in advanced NSCLC.

The deteriorated dyslipidemia caused by anlotinib might provide an explanation for the poor prognosis of the patients in the obesity group. The elimination half-life (t1/2) of anlotinib was considerably longer than other tyrosine kinase inhibitors (Li, 2021). Previous studies have reported that the decreased mRNA expression and activity of the main metabolic enzymes of anlotinib are found in obese individuals (Morrish et al., 2011; Zhong et al., 2018). Moreover, a significant inductive effect was not observed on the metabolic enzymes of anlotinib (Sun et al., 2017). Thus, obesity might lead to the accumulation of anlotinib, which might cause increased incidence of adverse events (AEs). Dyslipidemia is a common complication of obesity and one of the AEs of anlotinib (Han et al., 2018a; Vekic et al., 2019). An association between elevated pretreatment blood lipid and poor prognosis was observed in patients receiving anlotinib for advanced NSCLC (Poirier et al., 2006; Lijnen et al., 2007; Wells et al., 2012; Ebadi and Mazurak, 2014; Jang et al., 2016; Tang et al., 2021). Therefore, we supposed that the administration of anlotinib might cause worsened dyslipidemia and lead to a decreased survival time of the obese patients.

To our knowledge, this is the first time when a U-shaped relationship was found between BMI and efficacy of an anti-angiogenesis drug. Previous studies have reported that obesity is associated with improved OS in patients with lung cancer (Leung et al., 2011; Chen et al., 2021). In accordance with the conclusion, in our study, obese patients receiving a placebo have a median longer OS than the other three groups. Such a result might indicate that obese patients with lung cancer have an increased OS than those with a normal or low BMI. Nevertheless, the administration of anlotinib might lead to an inferior prognosis in this population. Thus, careful consideration should be given to the issue of whether obese patients need to receive anlotinib treatment for refractory NSCLC. However, to our best knowledge, this is the first time a U-shaped relationship was reported between BMI and effectiveness of anlotinib in advanced NSCLC patients. What is more, the sample size in the obese group was quite small in our study. Therefore, the finding must be interpreted with caution.

Although the study about the relationship between obesity and the effectiveness of anlotinib was still absent, the association of BMI and the efficacy of bevacizumab, another anti-angiogenesis drug, has been investigated before. Previous studies have suggested that elevated BMI was associated with poor prognosis in patients receiving bevacizumab for advanced CRC (Faruk Aykan et al., 2013) and epithelial ovarian cancer (Slaughter et al., 2014), whereas the association of increased BMI with improved OS was observed in advanced CRC patients treated with chemotherapy rather than those with the combination of chemotherapy and bevacizumab (Simkens et al., 2011). Hence, it is still controversial whether and how high BMI influences the prognosis of patients treated with anti-angiogenesis drugs for different cancers. Further investigation is clearly warranted.

Our result demonstrated that BMI was an independent predictor of survival for advanced NSCLC patients treated with anlotinib. Anlotinib is an inhibitor targeting multiple molecules included in tumor progression, which has been approved in NSCLC patients having disease progression after two or more lines of chemotherapy (Zhou et al., 2019). Compared with other potential predictors (Lu et al., 2019a; Lu et al., 2019b; Lu et al., 2019c), BMI is more available, convenient, and with acknowledged classification. Thus, BMI might be a promising predictive factor for anlotinib treatment.

There are some limitations in our study. First, the study was limited by its retrospective nature. A previous study has demonstrated that weight change during treatment provided more important information than a simple comparison of pre-treated BMI (Patel et al., 2016). Nevertheless, post-treatment BMI values were not available in our study. Second, the sample size of our study was relatively small, which might cause the considerable difference of the patient numbers in the non-obesity group and the obesity group. Thus, the result must be interpreted with caution. Third, it is controversial whether BMI is an appropriate measure of obesity (Griggs and Sabel, 2008). The predictive role of other body composition measures has been reported in cancer risk, such as waist circumference, waist-to-hip ratio (Pischon et al., 2006), and visceral fat area (Guiu et al., 2010; Patel et al., 2015). Hence, our results need further validation in prospective studies based on a larger population.

## Conclusion

Our findings revealed a U-shaped relationship between BMI and risk of death in patients receiving anlotinib for advanced NSCLC. More importantly, the interaction between BMI classification and treatment was significant for OS. Therefore, obesity (BMI ≥28 kg/m^2^) might be a poor predictor of the efficacy of anlotinib in NSCLC.

### Clinical Practice Points

The relationship between obesity and favorable prognosis has been reported in lung cancer patients, which is called the “obesity paradox”. In non-small cell lung cancer (NSCLC) patients receiving epidermal growth factor receptor tyrosine kinase inhibitor (TKI), better prognosis has been observed in overweight and obese patients. As a novel TKI, anlotinib suppresses tumor proliferation by inhibiting tumor angiogenesis. Some studies have demonstrated that elevated body mass index (BMI) is associated with poor prognosis in metastatic colorectal cancer patients receiving anti-angiogenesis treatment. However, it remains unclear whether increased BMI is associated with poor efficacy of anlotinib in NSCLC. In this study, our results revealed a U-shaped relationship between BMI and risk of death in patients receiving anlotinib for NSCLC. More importantly, obesity (BMI ≥28 kg/m^2^) might be a poor predictor of the efficacy of anlotinib in NSCLC.

## Abbreviations

BMI, body mass index; CR, complete response; DCR, disease control rate; ECOG, Eastern cooperative oncology group; EGFR-TKI, epidermal growth factor receptor; NSCLC, non-small cell lung cancer; PFS, progression-free survival; OS, overall survival; ORR, objective response rate; mCRC, metastatic colorectal cancer; PR, partial response; SD, stable disease; RCS, restricted cubic spline; HRs: hazard ratios; PS, performance status; VEGFR, vascular endothelial growth factor receptor.

## Data Availability

Publicly available datasets were analyzed in this study. This data can be found here: 10.1038/bjc. 2017.478 10.1001/jamaoncol. 2018.3039.
