# Supplementary material for: Association Between Obesity and Poor Prognosis in Patients Receiving Anlotinib for Advanced Non-Small Cell Lung Cancer
Source: Front Pharmacol. 2022 Mar 30;13:812555. doi: 10.3389/fphar.2022.812555 (PMC9005904; doi:10.3389/fphar.2022.812555)
Supplement: Supplementary file 1 [file Table1.docx]

**Supplementary Table 1**. Summary of secondary endpoints in non-obesity group.

|  | Anlotinib (n=318) | Placebo (n=187) | HR (95% CI) | *P* |
| --- | --- | --- | --- | --- |
| **Progression-free survival** | | | | |
| Patients with event (%) | 231 (72.6) | 153 (81.8) | -- | -- |
| Median (months, 95% CI) | 4.8 (4.2-5.4) | 1.3 (1.2-1.5) | 0.20 (0.16-0.26) | **<0.001** |
| **Objective response rate** | | | | |
| Patients with event (%) | 32 (10.1) | 1 (0.5) | -- | **<0.001** |
| **Disease control rate** | | | | |
| Patients with event (%) | 258 (81.1) | 65 (34.8) | -- | **<0.001** |

The bold values mean that the p value was considered statistically significant.

HR, hazard ratio; CI, confidence interval.
