# Supplementary material for: Association Between Obesity and Poor Prognosis in Patients Receiving Anlotinib for Advanced Non-Small Cell Lung Cancer
Source: Front Pharmacol. 2022 Mar 30;13:812555. doi: 10.3389/fphar.2022.812555 (PMC9005904; doi:10.3389/fphar.2022.812555)
Supplement: Supplementary file 2 [file Table2.docx]

**Supplementary Table 2**. Summary of secondary endpoints in obesity group.

|  | Anlotinib (n=36) | Placebo (n=13) | HR (95% CI) | *P* |
| --- | --- | --- | --- | --- |
| **Progression-free survival** | | | | |
| Patients with event (%) | 28 (77.8) | 7 (53.8) | -- | -- |
| Median (months, 95% CI) | 5.5 (3.9-7.1) | 1.4 (0-3.1) | 0.24 (0.07-0.84) | **0.025** |
| **Objective response rate** | | | | |
| Patients with event (%) | 1 (2.8) | 0 (0) | -- | 1.000 |
| **Disease control rate** | | | | |
| Patients with event (%) | 29 (80.6) | 6 (46.2) | -- | **0.046** |

The bold values mean that the p value was considered statistically significant.

HR, hazard ratio; CI, confidence interval.
